# Mean platelet volume as an inflammation marker in active pulmonary tuberculosis

**DOI:** 10.1186/2049-6958-9-11

**Published:** 2014-02-28

**Authors:** Gulsah Gunluoglu, Esra Ertan Yazar, Nurdan Simsek Veske, Ekrem Cengiz Seyhan, Sedat Altin

**Affiliations:** 1 Yedikule Teaching Hospital for Chest Diseases and Thoracic Surgery, Istanbul, Turkey; 2 Chest Diseases, Medipol University, Istanbul, Turkey

**Keywords:** Inflammation markers, Mean platelet volume, Pulmonary tuberculosis

## Abstract

**Background:**

The mean platelet volume (MPV) reflects the size of platelets. It has been shown to be inversely correlated with level of the inflammation in some chronic inflammatory diseases. This prospective study aims to show the usability of MPV as an inflammation marker in patients with active pulmonary tuberculosis (PTB) by comparison with healthy controls. In addition, its relationships with other inflammatory markers such as C-reactive protein (CRP) and the erythrocyte sedimentation rate (ESR) as well as with the radiological extent of disease were examined.

**Methods:**

This study included 82 patients with active PTB and 95 healthy subjects (control group). Whole blood counts, CRP level, and ESR were compared between the two groups. In the PTB group, the relationships between the radiological extent of disease and the MPV and other inflammation markers were investigated.

**Results:**

The MPV was 7.74 ± 1.33/μL in the PTB group and 8.20 ± 1.13/μL in the control group (p = 0.005). The blood platelet count, CRP level, and ESR were significantly higher in the active PTB group than in the control group (p < 0.0001). In the PTB group, CRP levels (r = 0.26, p = 0.003) and ESR (r = 0.39, p = 0.003), but not MPV (p = 0.80), were significantly correlated with the radiologic extent of the disease.

**Conclusions:**

The MPV was lower in patients with PTB than in healthy controls, however, the difference was limited. The MPV does not reflect the severity of the disease. The use of MPV as an inflammation marker and a negative acute-phase reactant in PTB does not seem to be reliable.

## Background

Tuberculosis (TB) is a common infectious disease caused by *Mycobacterium tuberculosis* (Mtb), and it remains an important public health problem despite developments in its diagnosis and treatment. According to a World Health Organization report, TB caused the death of 1,4 million people worldwide in 2010 [1]. Delayed diagnosis and treatment are also important public health issues. The only definitive diagnostic method known for pulmonary TB (PTB) is the growth of Mtb bacilli from pulmonary material (sputum or bronchoscopic lavage fluid) [2]. Although no other current test is more reliable for determining the activity of PTB than culture growth of bacilli, some biochemical parameters that reflect the inflammation present in patients with this condition may provide guidance at the diagnostic stage.

TB is an infectious disease characterised by a cellular immune response. Mtb and its components activate macrophages and lymphocytes, which then secrete cytokines (TNF-α, IL-6, IL-8, and IL-12) enabling the development of a cellular immune response [3,4]. Of these cytokines, TNF-α and IL-6 in particular affect maturation of thrombopoietic cells and secretion of platelets into the circulation [5]. The CRP level, ESR, and presence of reactive thrombocytosis can also reportedly be used to determine the disease activity, and these values are correlated with the disease severity [6-8].

The mean platelet volume (MPV) reflects the size of platelets. It can be measured during a routine automatic whole blood count. The MPV has been shown to correlate with the function and activation of platelets [9-11]. The importance of MPV has been emphasized as an inflammation marker in some chronic inflammatory disorders, such as inflammatory intestinal diseases, rheumatoid arthritis, and ankylosing spondylitis. An inverse correlation between disease activity and MPV has been demonstrated [12-15].

This study aims to show the usability of the MPV as a readily obtainable negative inflammation marker that can be measured during a routine whole blood count for assessment of disease activity in patients with active PTB by comparison with healthy controls. This study also investigates the relationships between MPV and other inflammatory markers (ESR and CRP level) as well as the radiological extent of the disease.

## Methods

This prospective study was performed in accordance with the principles of the Declaration of Helsinki and approved by the Ethics Committee of Yedikule Chest Diseases and Thoracic Surgery Training and Research Hospital.

Patients who presented to the outpatient clinic of our hospital between June 2013 and September 2013 and were diagnosed with PTB based on clinical symptoms, radiological signs, and examination of sputum smears and cultures for Mtb were included in the study. Healthy subjects who presented to the outpatient clinic for routine examination, and presented no disease and normal lung radiography findings during the examination, were included in the study as control group.

The exclusion criteria were: age < 18 or > 60 years, acute or chronic infection, systemic arterial hypertension, endocrinological disorders, haematological disease, heart failure, hepatic and renal disorders, cancer, and peripheral vascular disease [16]. None of the enrolled subjects had received anticoagulant medications, nonsteroidal anti-inflammatory drugs, or contraceptives.

Detailed histories of all patients and healthy subjects were obtained, and their smoking status were noted. A physical examination of all participants was performed, and weights and heights were measured to calculate their body mass index (BMI). Chest X-rays were also performed. Venous blood was drawn from all participants (pre-treatment samples in patients with PTB). The blood samples were placed in tubes containing EDTA. A whole blood count analysis was carried out within 120 min to assess the MPV (μL) [17]. The Cell-Dyn 3700 haematologyanalyser (Abbott Laboratories) was used for this analysis. The values obtained at the end of the analysis were recorded. Serum CRP levels (normal, 0–5 mg/L) were measured using an Olympus AU2700 plus analyser (Beckman Coulter, Tokyo, Japan), and the ESR (60 min) was determined using an Eriline AR analyser (Linear Chemicals, Barcelona, Spain).

The disease was categorised according to its radiological extent in patients with PTB based on the criteria by Dlugovitzky et al. [18]. Accordingly, the extent was classified as mild disease (single-lobe involvement and no visible cavities or pleural involvement), moderate disease (unilateral involvement of two or more lobes with a solitary cavity, or mild extent plus pleural involvement or effusion), or severe disease (bilateral involvement and multiple cavities, or moderate extent plus pleural involvement or effusion).

The blood platelet counts, MPVs, serum CRP levels, and ESRs in the patient and healthy groups were compared. In addition, the relationship between the radiological extent of disease and inflammation markers were assessed in the patient group.

### Statistics

The data were entered into the SPSS analysis software. A chi-square test was used to compare frequencies, and Student’s t-test was used to compare averages. Pearson’s correlation test was used to show correlations between continuous variables, and Spearman’s correlation test was used for correlation analyses involving non-parametric variables.

## Results

The study group comprised 82 patients with active PTB and 95 healthy subjects. The participants’age, sex, smoking habits, and BMI are shown in Table 1. The mean age, sex distribution, and number of individuals with smoking habits showed no statistically significant differences between the two groups. The BMI was lower in patients with active PTB than in the healthy controls, and the difference was statistically significant. No significant correlation was found between MPV and BMI (p = 0.103). The MPV was significantly lower in the active PTB group than in the control group (p = 0.005).

**Table 1:** Comparison of age, sex, smoking habit and BMI values of healthy subjects and tuberculosis patients

	**Healthy subjects**	**Tuberculosis patients**	**P value**
Gender, n (male/female)	65/30	54/28	>0.05
Age (median±SD)	36±9.96	26.5±1.16	0.05
Smokers n (%)	39 (41.1%)	44 (53.7%)	>0.05
BMI (median±SD)	24±3.5	20.6±2.8	<0.0001

BMI; Body mass index, SD; standard deviation.

The levels of the other investigated inflammatory markers also differed between the two groups (Table 2).

**Table 2:** Comparison of level of MPV and the other inflammatory markers in the healthy subjects and tuberculosis patients groups

	**Healthy subjects**	**Tuberculosis patients**	**P value**
MPV (mean±SD)	8.2±1.13	7.74±1.33	0.005
PLT (mean±SD)	269±70.50	404±146.70	<0.0001
CRP (mean±SD)	10.06±12.70	64.68±53.82	<0.0001
ESR (mean±SD)	2.47±3.51	54.47±28.69	<0.0001

MPV; mean platelet volume, PLT; platelet count, CRP; C-reactive protein, ESR; erythrocyte sedimentation rate, SD; standard deviation.

The correlation between MPV and platelet count was analysed. In patients with PTB, there was an inverse relationship between these parameters, but it was not statistically significant (p = 0.8). In the healthy control group, on the other hand, there was a moderate (r = 0.25) and statistically significant (p = 0.015) inverse correlation between MPV and platelet count.

No significant relationship was found between the radiological extent of disease and the platelet count in patients with PTB (p = 0.24). Also, no correlation was found between the MPV and radiological extent of disease (p = 0.80). There was a moderate and significant correlation between platelet count and CRP level (r = 0.60, p = 0.0001) and ESR (r = 0.64, p = 0.0001). Additionally, there were significant correlations between the radiological extent of disease and CRP level (r = 0.26, p = 0.003) and ESR (r = 0.39, p = 0.003).

## Discussion

Active TB has been associated with an imbalance of the Th1/Th2 cytokine pattern. Effective induction of Th1 immunity is vital in the defence against Mtb. The major cytokines secreted in response to Mtb are IL-2, IFN-gamma, IL-6, IL-12, and TNF-α [19,20]. Animal models have demonstrated the presence of cytokines in TB granulomas. In addition, human studies have shown IL-1 beta, IL-6, and TNF-α, which are secreted from active macrophages in the bronchoalveolar lavage fluid of patients with active TB [21]. Nevertheless, a systemic inflammatory response is known to occur in patients with TB when these cytokines enter the systemic circulation, and as a result the CRP blood level and ESR increase.

Platelets are pulmonary immune cells as shown by their role in the pathogenesis in some pulmonary diseases and their immune cell characteristics [22,23]. In patients affected by PTB microthromboses form in vessels around the tuberculous cavities, probably as a defence mechanism [24]. These thromboses prevent the spread of Mtb, thus averting the occurrence of disseminated disease. Reactive thrombocytosis is seen in many chronic inflammatory diseases, including TB [7,25,26]. The increased number of platelets in patients with PTB has also been linked to the disease extent, which supports the above pathological changes [27,28].

The MPV reflects the size of platelets and is a marker used to determine platelet function. The size of platelets in the circulation is associated with the intensity of inflammation [16]. In some chronic inflammatory disorders, the MPV has been used as an inflammatory marker of disease activity and to monitor the response to anti-inflammatory treatment [12,15,29]. In acute exacerbations of chronic obstructive pulmonary disease, in which the intensity of inflammation also increases, the MPV has been found to be significantly lower while the serum leukocyte count and neutrophil percentage were higher than those during the stable period. It has been suggested that the MPV can be used as a negative acute-phase reactant [30]. When various cytokines, including IL-6, enter the systemic circulation in patients with TB, systemic symptoms emerge and an acute-phase response occurs [4,7,31]. In such cases of TB, the lower MPV can be explained by the fact that megakaryopoiesis is affected by the excess production of proinflammatory cytokines and acute-phase reactants which decreases the size of platelets, therefore smaller platelets are released from the bone marrow [9]. Similarly, Baynes et al. [25] found the MPV to be low in patients with active PTB and suggested that although thrombopoiesis increased in patients with TB, the platelets’ lifetime may have been shortened. Platelets and their indices in active PTB have been addressed in several previous studies [6,26,27,32]. Contrary to our study, Tozkoparan et al. [6] found that the MPV was significantly higher in patients with active TB than in control patients with inactive TB and non-specific pneumonia and decreased with anti-TB treatment. On the other hand, Sahin et al. [27] showed that in patients with active TB MPV was identical to that of patients with non-specific pneumonia and healthy subjects. There was no correlation between MPV and radiological extent of TB in a study of Sahin et al. as in our work. However, Tozkoparan et al. found a weak correlation. Disease-specific characteristics and cardiovascular risk factors (diabetes, hypertension, smoking, obesity, etc.) may directly affect the MPV [16]. This is why participants with risk factors were excluded from the study. In the above mentioned studies there were not enough data about the patients’ characteristics that could have affected the MPV. Contradictory results may be related to the characteristics of the studied groups of patients. An association has been established between obesity and high MPV, and a positive correlation has been demonstrated between BMI and MPV [33,34]. As expected, in our study BMIin the PTB patients was lower than in healthy controls but no relationship was found between BMI and MPV (p = 0.103).

CRP is an acute-phase reactant secreted from hepatocytes in response to tissue damage or inflammation. It has been shown to increase in proportion to the severity of TB and decrease after treatment [35]. It has also been linked to mortality [36]. The ESR, another acute-phase reactant, has been found to be high in patients with active PTB and to have a correlation with the radiological extent of disease [8]. Our study also demonstrated that the CRP level and ESR increased significantly in active disease and had a positive correlation with the radiological extent of the disease.

## Conclusions

In conclusion, in our study the MPV, measured automatically during a whole blood count, in patients with active PTB was lower than in healthy controls. However, the difference between PTB patients and healthy controls is limited and smaller than the difference between CRP levels or ESRs of patients and those of healthy controls. The MPV had no correlation with thrombocytosis, acute-phase reactants or the radiological extent of the disease. Therefore, it does not reflect the disease severity. MPV does not seem to be reliable as an inflammation marker to determine the disease activity in patients with PTB and as a negative acute-phase reactant. Thus, further prospective studies on this issue with the inclusion of larger numbers of patients are mandatory.

## Availability of supporting data

The dataset supporting the results in the submitting manuscript is not available in a publicly-accessible data repository.

## Competing interests

There is no any financial or other manner support. The authors’ declare that they have no competing interests.

## Authors’ contributions

All Authors have been involved in drafting the manuscriptor revising. Each author have given final approvel of the version to be published and agree to be accountable for all aspects of the work.

Dr. GG, have made substantial contributions to conception and design, acquisition of data and writing of manuscript. Dr. EEY; made acquisition and analysis of data. Dr. NŞV, made acquisition of data, contrubutions to conception. Dr. ECS, made design of data, statistical analyzed. Dr. SA, have made substantial contributions to conception and design.
